# Lysosomal storage and impaired autophagy lead to inflammasome activation in Gaucher macrophages

**DOI:** 10.1111/acel.12409

**Published:** 2015-10-21

**Authors:** Elma Aflaki, Nima Moaven, Daniel K. Borger, Grisel Lopez, Wendy Westbroek, Jae Jin Chae, Juan Marugan, Samarjit Patnaik, Emerson Maniwang, Ashley N. Gonzalez, Ellen Sidransky

**Affiliations:** ^1^ Section of Molecular NeurogeneticsNational Human Genome Research InstituteBethesdaMD20892USA; ^2^ Inflammatory Disease SectionNational Human Genome Research InstituteBethesdaMD20892USA; ^3^ National Center for Advancing Translational ScienceNational Institutes of HealthBethesdaMD20892USA

**Keywords:** autophagy, Gaucher disease, glucocerebrosidase, inflammasome, interleukin‐1β, lysosome, macrophage

## Abstract

Gaucher disease, the inherited deficiency of lysosomal glucocerebrosidase, is characterized by the presence of glucosylcer‐amide macrophages, the accumulation of glucosylceramide in lysosomes and the secretion of inflammatory cytokines. However, the connection between this lysosomal storage and inflammation is not clear. Studying macrophages derived from peripheral monocytes from patients with type 1 Gaucher disease with genotype N370S/N370S, we confirmed an increased secretion of interleukins IL‐1β and IL‐6. In addition, we found that activation of the inflammasome, a multiprotein complex that activates caspase‐1, led to the maturation of IL‐1β in Gaucher macrophages. We show that inflammasome activation in these cells is the result of impaired autophagy. Treatment with the small‐molecule glucocerebrosidase chaperone NCGC758 reversed these defects, inducing autophagy and reducing IL‐1β secretion, confirming the role of the deficiency of lysosomal glucocerebrosidase in these processes. We found that in Gaucher macrophages elevated levels of the autophagic adaptor p62 prevented the delivery of inflammasomes to autophagosomes. This increase in p62 led to activation of p65‐NF‐kB in the nucleus, promoting the expression of inflammatory cytokines and the secretion of IL‐1β. This newly elucidated mechanism ties lysosomal dysfunction to inflammasome activation, and may contribute to the massive organomegaly, bone involvement and increased susceptibility to certain malignancies seen in Gaucher disease. Moreover, this link between lysosomal storage, impaired autophagy, and inflammation may have implications relevant to both Parkinson disease and the aging process. Defects in these basic cellular processes may also provide new therapeutic targets.

**Abbreviations**
GD: Gaucher disease
GCase: glucocerebrosidase
GluCer: glucosylceramide
GMs: Gaucher macrophages
PRR: pathogen recognition receptor
TLRs: Toll‐like receptors
NLR: NOD‐like receptors
PAMPs: pathogen‐associated molecular patterns
DAMPs: danger‐associated molecular patterns
ASC: apoptosis‐associated speck‐like proteins
VPS34: vacuolar protein sorting‐34
PE: phosphatidylethanolamine
LC3: microtubule‐associated protein‐light chain 3
LPS: lipopolysaccharide
PBMCs: peripheral blood monocytes
IL: interleukin
ELISA: enzyme‐linked immunoassay
Baf: bafilomycin A1
Rapa: rapamycin
3MA: 3‐methyladenine

## Introduction

In Gaucher disease (GD), the most common lysosomal storage disorder, macrophages accumulate glycolipids within lysosomes due to the defective digestion of aged erythrocytes and senescent leukocytes (Lee, 1968). The disease results from a deficiency of the lysosomal enzyme glucocerebrosidase (GCase), which cleaves a glucose moiety from the glycolipid glucosylceramide (GluCer). This lysosomal storage leads to the characteristic engorged macrophages, known as Gaucher macrophages (GMs) or ‘Gaucher cells’ (Machaczka *et al*., 2011), and contributes to the organomegaly, bone disease and cytopenia typically encountered in patients with GD. Furthermore, glycolipid accumulation and lysosomal dysfunction in GD serve to prime macrophages to release pro‐inflammatory cytokines in response to stimuli (Michelakakis *et al*., 1996; Allen *et al*., 1997; Barak *et al*., 1999). Thus, GMs provide a unique model to explore the mechanisms underlying defective erythrophagocytosis and its relationship to inflammation.

Macroautophagy is a proteolytic process whereby organelles and cellular proteins are encapsulated and delivered to lysosomes (Mizushima & Komatsu, 2011). This form of autophagy takes place in three phases: induction, autophagosome formation, and degradation. Induction of autophagy is stimulated by pathogen recognition receptor (PRR) ligands, and plays a critical role in the capture and degradation of pathogens (Xu & Eissa, 2010). After the induction of autophagy, vesicle nucleation begins with the assembly of phagophores, lipid membranes organized by a protein complex consisting of the lipid kinaselike vacuolar protein sorting‐34 (VPS34) and beclin‐1 (Kihara *et al*., 2001). This complex initiates the phosphorylation of phosphatidylinositol‐3‐phosphate, which leads to the formation of autophagophores. Autophagophores are elongated via an ubiquitin E3‐ligase complex that includes Atg7, Atg12, and Atg16L1, which conjugates phosphatidylethanolamine (PE) to microtubule‐associated protein‐light chain 3 (LC3). p62 binds to LC3‐PE, keeping it in the inner and outer membranes of autophagosomes. Autophagosomes then fuse with lysosomes to form autophagolysosomes, which act to degrade their contents either for recycling in the cell, or for presentation of as antigens (Franchi *et al*., 2012).

Macrophages undergo M1 (classical) or M2 (alternative) activation in response to different stimuli (Gordon & Martinez, 2010). M1 macrophages secrete pro‐inflammatory cytokines and chemokines, including interleukin (IL)‐1β, IL‐6, IL‐12, and CCL2 (Scotton *et al*., 2005). The secretion of pro‐inflammatory cytokines by macrophages is often a response to pathogens, and occurs by means of toll‐like receptors (TLRs) and NOD‐like receptors (NLRs). These receptors recognize pathogen‐associated molecular patterns (PAMPs) or danger‐associated molecular patterns (DAMPs) (Rathinam *et al*., 2012). In the case of NLRs, recognition of PAMPs or DAMPs leads to the formation of a signaling protein complex known as the inflammasome. Inflammasomes contain oligomerized NLRs, which bind to caspase‐1 either directly, or via any one of a group of adaptor proteins known as apoptosis‐associated speck‐like proteins (ASCs). Activation of caspase‐1 results in the processing of pro‐IL‐1β and pro‐IL‐18 to form mature IL‐1β and IL‐18, respectively. IL‐1β release requires two PRR signals; p65‐NF‐kB‐dependent expression of pro‐IL‐1β downstream of TLR activation, and caspase‐1‐mediated cleavage of pro‐IL‐1β downstream of NLR activation and inflammasome formation (Eder, 2009). Among the various NLRs, the NLRP3 inflammasome has been best described, and is activated by a wide range of DAMPs, including ATP, K+ efflux, particulate crystals, mitochondrial reactive oxygen species, and mitochondrial DNA (Nakahira *et al*., 2011). Recently, the regulation of inflammasomes by autophagy has been the subject of considerable attention (Carneiro & Travassos, 2013), and it has been shown in macrophages that upon TLR ligation, IL‐1β is sequestered into the autophagosome (Harris *et al*., 2011).

The association of inflammasomes and autophagy also has relevance for the field of aging. The aging process is associated with a deterioration in autophagic capacity and enhanced cellular stress, resulting in NLRP3 activation (Salminen *et al*., 2012). Furthermore, studying the link between lysosomal dysfunction and inflammation in GD may directly impact our understanding of a common neurodegenerative disorder of aging, Parkinson disease (PD). Mutations in the glucocerebrosidase gene (*GBA1)* are an important genetic risk factor for PD and related Lewy body disorders (Sidransky *et al*., 2009). A better understanding of the implications of lysosomal impairment and autophagy in GD may yield insights relevant to PD pathogenesis.

Research into the mechanism of lysosomal dysfunction in GMs has been impeded by the lack of appropriate cellular models that display the storage phenotype observed in patients. Moreover, GD is a phenotypically heterogeneous disease with more than 300 known mutations and limited correlation between genotype and phenotype. Here, using GMs derived from patients with the most common GD genotype (N370S/N370S), we demonstrate that autophagy is impaired as a result of this lysosomal storage. We then utilize this model to show that impaired autophagy triggers inflammasome activation and IL‐1β secretion.

Gaucher disease is often treated by enzyme replacement therapy using intravenously infused, recombinant GCase. However, its high cost and inability to cross the blood–brain barrier have prompted the search for chemical chaperones that can enhance GCase levels and reduce lysosomal storage in GD (Suzuki, 2013). We identified a noninhibitory chaperone molecule NCGC00188758, a pyrazolo (N‐(4‐ethynylphenyl)‐5,7‐dimethylpyrazolo[1,5‐a]pyrimidine‐3‐carboxamide) (Patnaik *et al*., 2012), referred to here as NCGC758, that can reverse the disease phenotype of GMs by facilitating the translocation of GCase from the endoplasmic reticulum to the lysosome (Aflaki *et al*., 2014). In this study, we show that this noninhibitory chaperone can induce autophagy and reduce IL‐1β secretion in GMs, confirming that deficient GCase accounts for the phenomena observed.

## Results

### Gaucher macrophages demonstrate increased release of inflammatory cytokines in response to lipopolysaccharide

**Figure 1:**
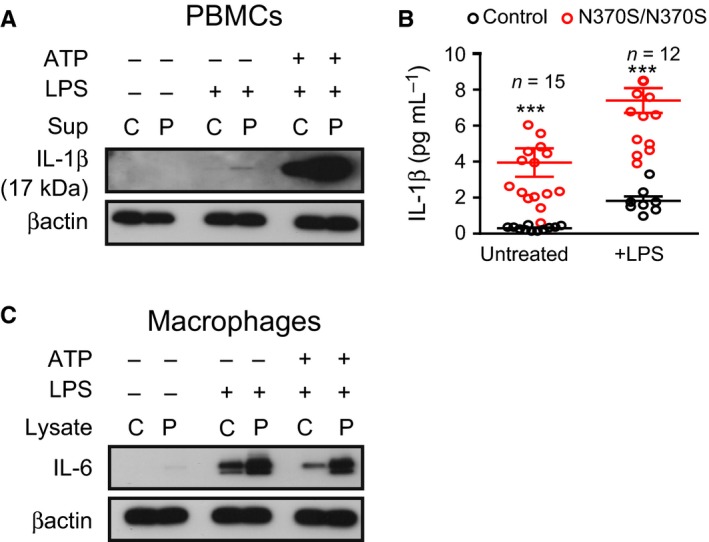
Gaucher macrophages show inflammasome activation and secrete IL‐1β (A) Control (C) and N370S/N370S (P) peripheral blood monocytes were treated with lipopolysaccharide (LPS) or LPS and ATP and the supernatant probed for IL‐1β. (B) An ELISA for activated IL‐1β was performed using the supernatants from patient and control macrophages treated with LPS. All samples were evaluated in duplicate by ELISA. n = number of patients. (C) Macrophage lysates were probed for IL‐6. Statistical significance; *P* < 0.05(*), *P* < 0.01(**), *P* < 0.001(***).

To evaluate the effect of lysosomal dysfunction on inflammasome activation (Schroder & Tschopp, 2010; Levine *et al*., 2011), we assessed IL‐1β secretion in both peripheral blood monocytes (PBMCs) (Fig. 1A) and macrophages (Fig. 1B,C) from patients with GD and controls by Western blots and ELISA. There was only modest secretion of IL‐1β in Gaucher and control PBMCs treated with LPS alone, but IL‐1β secretion increased significantly when LPS was supplemented with ATP, a potent activator of NLRP3. Moreover, after treatment with LPS, IL‐1β levels measured by ELISA were 7‐fold higher in GMs than in controls (Fig. 1B). In addition, increased levels of IL‐6 were detected in lysates from GMs treated with either LPS alone or LPS with ATP, compared to control macrophages (Fig. 1C). These findings suggest that NLRP3 activation is increased in GD, causing secretion of inflammatory cytokines IL‐1β and IL‐6 in response to LPS.

### Increased activation of inflammasomes in Gaucher macrophages is a consequence of lysosomal storage

**Figure 2:**
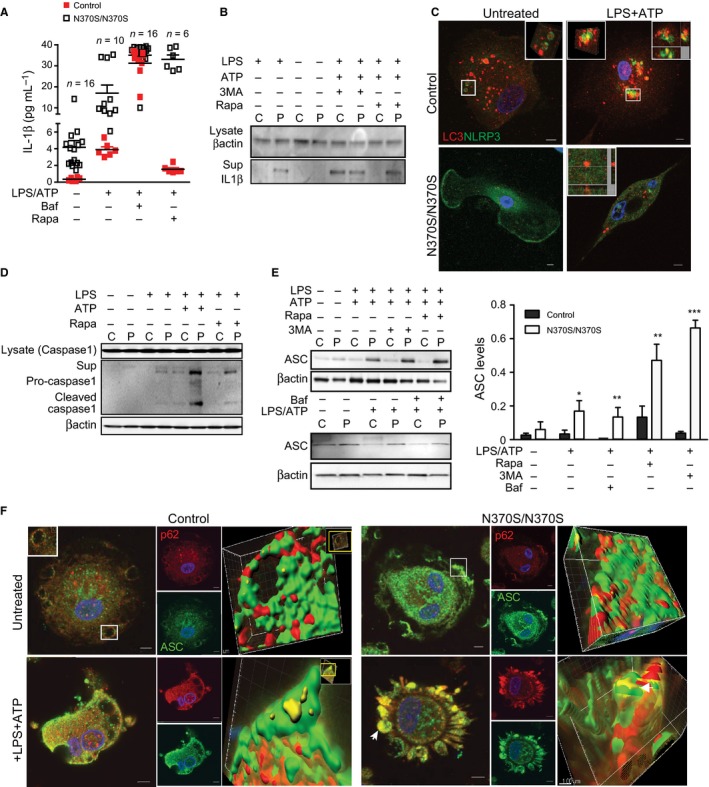
Caspase‐1 activation and increased ubiquitinated protein apoptosis‐associated speck‐like proteins (ASC) in Gaucher macrophages (GMs). (A) ELISA or (B) Total supernatants or total protein lysates from treated macrophages from patients (P) and controls (C) were probed for IL‐1β. (C) Control (C) and GMs (P) were immuno‐stained for LC3 (red) and NLRP3 (green) after treatment with lipopolysaccharide (LPS) (100 ng) and ATP (5 mm) for 1 h. Cells were imaged using a confocal microscope (Z‐stack with 0.5 μm thickness). Co‐localization was evaluated using Imaris software. Merged channels are shown as yellow. Pictures represent seven independent experiments performed on cells from seven different patients. Single channels are presented in the supplements. (D) Caspase‐1 was analyzed in total lysate and supernatant by immunoblotting, after treatment with LPS alone or LPS (100 ng)+ATP (5 mm) or rapamycin (25 nm). Data represent five independent experiments. (E) Total protein from control and Gaucher macrophages was immunoblotted for ASC after treatment with LPS (100 ng) and ATP (5 mm), and then rapamycin (25 nm), 3MA (5 mm) or Baf.1A (10 μm) for 40 min. ASC expression was normalized to β‐actin. The blot is representative of seven independent experiments performed on samples from seven different patients and controls. *P* < 0.05(*) and *P* < 0.01(**), *P* < 0.001(***). (F) Control and GMs were immunostained for p62 (red) and ASC (green) in the absence and presence of LPS (100 ng) and ATP (5 mm) and then imaged by confocal microscopy. Merged channel volumes are shown in yellow, and insets showing surface renderings of three‐dimensional reconstruction using Imaris software are shown in the far right panels.

Autophagy is reported to be involved in the regulation of the immune response to both pathogens and inflammation, and inhibition of autophagy induces inflammasome activation (Schroder & Tschopp, 2010; Levine *et al*., 2011). We examined inflammasome activation in the presence of chemicals that induce or inhibit autophagy. Immunoblotting and ELISAs were performed to assess IL‐1β processing. Both showed that inflammasome priming led to 9‐fold higher levels of mature IL‐1β in GMs compared to control macrophages, while suppressing autophagy with 3MA or Baf.A1 resulted in increased activated IL‐1β in both (Fig. 2A,B). Importantly, adding rapamycin after LPS and ATP treatment inhibited IL‐1β activation in control macrophages, but did not prevent IL‐1β secretion by GMs (Fig. 2A,B).

Next, the processing of inflammasomes by autophagosomes was evaluated in GMs. Immunostaining for both the inflammasome markers NLRP3 and endogenous LC3 demonstrated co‐localization in control, but not GMs, after inflammasome stimulation with LPS and ATP, reflecting impaired engulfment of inflammasomes by autophagosomes in GMs (Figs 2C and S1A,B). To evaluate inflammasome activation, we measured activated caspase‐1 in the lysates and culture supernatants from control macrophages and GMs in the presence or absence of LPS or LPS and ATP (Fig. 2D). With LPS stimulation alone, activated caspase‐1 was observed in GMs, but not control macrophages. Adding ATP induced caspase‐1 activation in both control macrophages and GMs, although levels were significantly higher in GMs. We then induced autophagy by adding rapamycin after LPS treatment. Caspase‐1 remained activated in GMs after treatment with rapamycin, although to a lesser extent than with LPS and ATP, while rapamycin inhibited caspase‐1 activation in control macrophages. This finding reflects persistent activation of inflammasomes in GMs, even with augmentation of autophagy by rapamycin.

### Increased SQMT1/p62 in Gaucher cells suppresses autophagy and induces inflammasome activation

The signaling adaptor p62 functions to deliver ubiquitinated substrates to the autophagosome. As ASC, the adaptor protein involved in the assembly of the inflammasome complex, is polyubiquitinated, it has been proposed that aggregates of inflammasomes could be targeted to the autophagic pathway by p62 (Shi *et al*., 2012). Immunoblotting for ASC, we observed that at baseline, ASC levels were 2‐fold higher in GMs than control macrophages (Fig. 2E). Subsequent induction of autophagy with rapamycin after LPS and ATP treatment resulted in decreased ASC levels in control macrophages, but not in GMs. Immunostaining of cells treated with LPS alone showed that only GMs had significant co‐localization of ASC with p62 (Fig. S1C). However, when ATP was added, both control macrophages and GMs showed co‐localization of ASC with p62, demonstrating that polyubiquitinated ASC could be targeted to the autophagic pathway by p62 (Fig. 2F).

**Figure 3:**
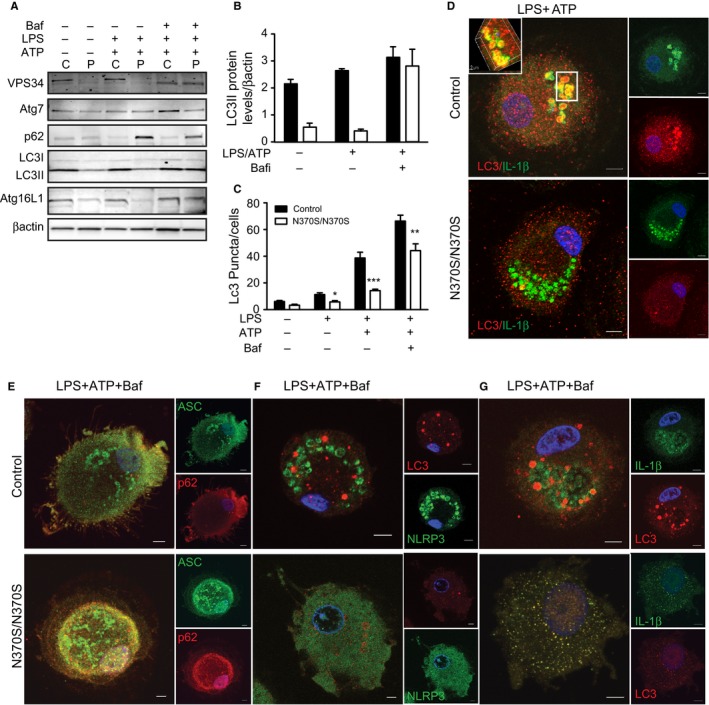
Impaired autophagy results in activation of inflammasomes in Gaucher macrophages (GMs). (A) Control (C) and GMs (P) (*n* = 4) were treated with lipopolysaccharide (LPS) (100 ng) or LPS+ATP (5 mm). 10 μm Baf.1A was added for 40 min after stimulation with LPS + ATP. Total lysates were run on SDS‐PAGE gels, and blots were probed with antibodies to VP34, Atg7, p62, LC3, Atg 16L1, and β‐actin. (B) LC3II protein levels normalized to β‐actin. (C) Results of a puncta assay performed in control and GMs in four independent experiments. Graph reflects the number of LC3 puncta counted per cell. Control and GMs, treated as described above, were then immunostained for (D) LC3 (red) and IL‐1β (green). (E) p62 (red) and apoptosis‐associated speck‐like proteins (green), (F) NLRP3 (green) and LC3 (red) and (G) IL‐1β (green) and LC3 (red). Images represent 20 pictures taken in 5 independent experiments (63X magnification and scale bar; 5μ).

We then studied whether the inhibition of autophagy also reduces inflammasome degradation in the cytosol. When cells were treated with LPS and ATP, VPS34 levels were lower in GMs than controls, but the difference was not seen when autophagy was suppressed with Baf.1A (Fig. 3A). Furthermore, in untreated and LPS‐treated cells, Atg7 levels were similar in both GMs and control macrophages. Blocking autophagy with Baf.1A followed by inflammasome activation increased levels of Atg7 in control, but not GMs. LC3I and LC3II are both processed post‐translationally, but LC3I is cytosolic and LC3II is membrane‐bound. We found that during inflammasome activation, LC3II levels were significantly higher in control than GMs, indicating enrichment of autophagosomes. Suppressing autophagy with Baf.A1 after inflammasome activation resulted in similar LC3II levels in both groups (Fig. 3A,B). In addition, when GMs were treated with Baf.A1 followed by LPS and ATP, there was a reduced number of LC3 puncta compared to control macrophages (Figs 3B,C and S2). Levels of Atg16L1 were lower in GMs, while p62 levels were significantly higher, both in the presence or absence of LPS and ATP. When Baf.1A was added, p62 levels remained higher in GMs than controls, while Atg16L1 was elevated in both (Fig. 3A). To confirm the role of autophagy in the regulation of inflammation, immunostaining for IL‐1β and LC3 was performed. Figure 3D shows that after inflammasome activation, IL‐1β co‐localized with LC3 in control macrophages, but not in GMs. This indicates that GMs have impaired maturation of autophagosomes. Immunostaining for ACS and p62, performed on control and GMs, showed that inhibiting autophagy after treatment with LPS and ATP increased co‐localization of ASC and p62 in both, indicating that activation of the inflammasome occurs in the cytosol (Fig. 3E). Under the same conditions, the lack of co‐localization between NLRP3 and LC3 indicates that inflammasomes were not present in the autophagosome (Fig. 3F). Staining with LC3 and IL‐1β after treatment with LPS and ATP and then Baf.1A resulted in co‐localization of LC3 and IL‐1β in both control and GMs, reflecting the accumulation of IL‐1β in autophagosomes (Fig. 3G). Together, these data indicate that activation of the inflammasome and IL‐1β secretion occur even after the induction of autophagy in GMs, providing further evidence of impaired autophagy in GD. In both control and GMs primed with LPS, the inhibition of autophagosome formation with 3MA, or the inhibition of autophagosome degradation with Baf.1A, resulted in secretion of IL‐1β, although there was significantly more IL‐1β secretion in GMs after induction of autophagy with rapamycin (Fig. 2A,B).

### Treatment of Gaucher macrophages with NCGC758 results in reduced IL‐1β secretion

**Figure 4:**
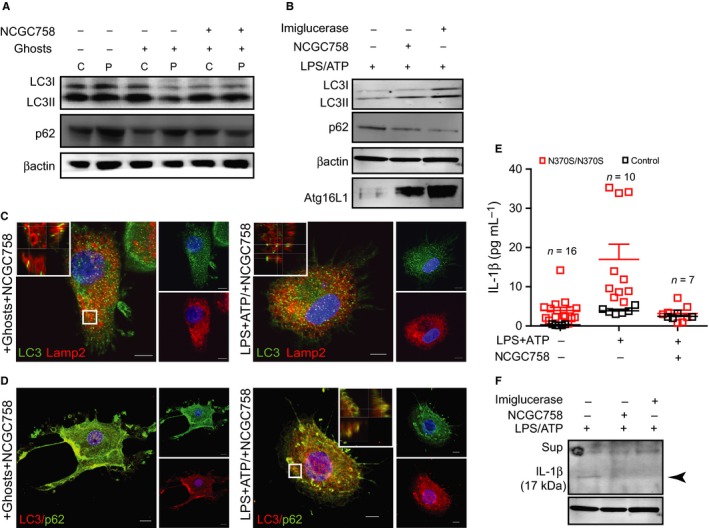
Treatment of Gaucher macrophages (GMs) macrophages with the small‐molecule NCGC758 results in reduced IL‐1β secretion (A) Control (C) and GMs (P) were treated with NCGC758 (8 μm) in the presence and absence of Gaucher erythrocyte ghosts. Total lysates were immunoblotted and probed for LC3 and p62. (B) Gaucher cells were treated with NCGC758 or imiglucerase (20 μm) followed by LPS+ATP (5 mm). Total protein lysates were run on SDS‐PAGE and were probed for LC3, P62, and Atg16L1. Blots represent two independent experiments. (C,D) Gaucher macrophages treated with NCGC758 in the presence of erythrocyte ghosts, or treated with LPS and ATP, were immunostained for (C) LC3 (green) and Lamp2 (red) and (D) LC3 (red) and p62 (green). Z‐stack images were acquired using a Zeiss 510 confocal microscope (63× magnification). Insets corresponding to the regions marked show higher magnifications of the areas outlined with the position of the *xy*‐, *xz*‐, and *yz*‐slices that are shown for each of the 3D stacks. Images are taken at the same laser settings and are representative of three independent experiments (scale bar; 5μ). (E,F) Control (C) and GMs (P) were treated with NCGC758 (8 μm) or Imiglucerase in the presence and absence of LPS+ATP and the supernatants (E) or total protein lysates (F) were probed for IL‐1β. Blots represent two independent experiments. n = number of patients.

In a recent study, we showed that treatment of GMs with the noninhibitory small‐molecule NCGC758 enhances translocation of GCase to the lysosome, resulting in significantly reduced levels of glucosylcer‐amide after erythrophagocytosis. Cells were fed with erythrocyte ghosts prepared from patients with GD to model the lipid storage seen in GMs *in vivo* (Aflaki *et al*., 2014). We studied the effect of NCGC758 on autophagy in treated GMs following erythrophagocytosis or inflammasome activation in the presence of LPS and ATP. Figures 4A and S3A,B indicate that treatment of GMs with NCGC758 leads to increased levels of LC3II after erythrophagocytosis, yet decreased levels of p62.

In addition, macrophages were treated with NCGC758 followed by LPS and ATP to activate the inflammasome (Figs 4B and S3C). NCGC758‐treated GMs showed increased LC3II and Atg16L1 and reduced p62 protein levels following inflammasome activation (Fig. 4B), which was also observed when the cells were treated with recombinant GCase (imiglucerase) (Fig. 4B).

Gaucher macrophages fed with erythrocyte ghosts and subsequently treated with NCGC758 were stained for the lysosomal marker Lamp2 and LC3 (Fig. 4C). Confocal microscopy showed that NCGC758 treatment results in co‐localization of Lamp2 with LC3 in GMs, demonstrating autophagosome maturation and the fusion of lysosomes and autophagosomes. Co‐localization of Lamp2 with LC3 was also observed in GMs pretreated with NCGC758 and then incubated with LPS and ATP to induce the inflammasome. Autophagosome maturation in NCGC758‐treated GMs was confirmed by co‐localization of p62 and LC3 during erythrophagocytosis and with LPS and ATP treatment (Fig. 4D).

Importantly, treatment of GMs with NCGC758 or imiglucerase followed by LPS and ATP also resulted in reduced IL‐1β secretion (Fig. 4E,F), which suggests that both treatments restored the autophagic capacity of GMs. These findings are confirmed that the autophagic impairments observed were indeed a result of GCase deficiency and the resultant lysosomal storage, both of which are restored after NCGC758 treatment (Aflaki *et al*., 2014).

### p65‐NF‐kB activation in Gaucher macrophages leads to secretion of inflammatory cytokines

**Figure 5:**
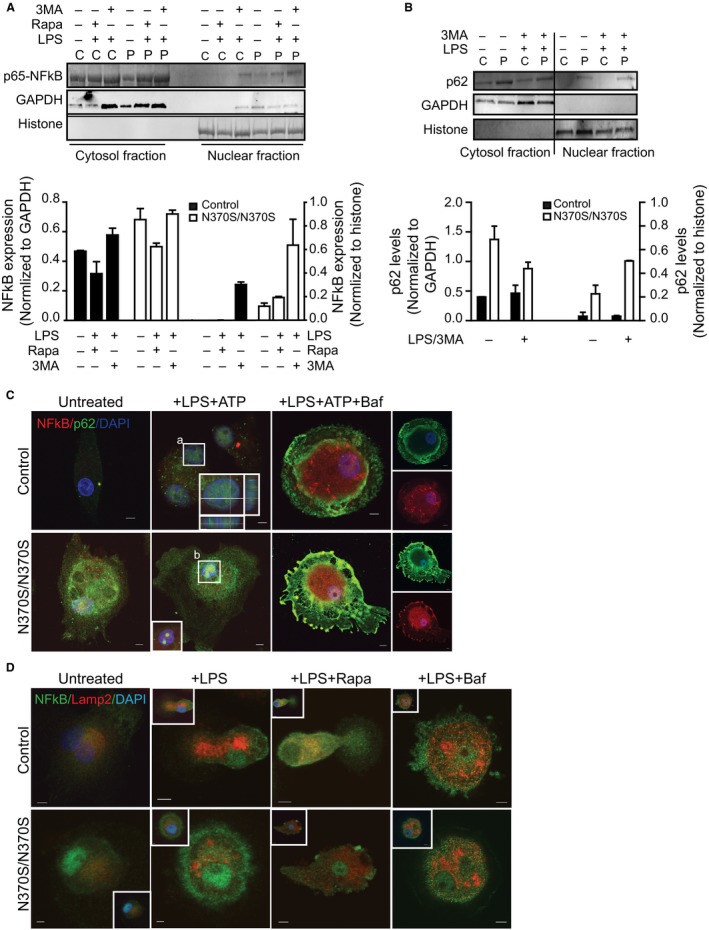
p65‐NF‐kB activation in Gaucher macrophages (GMs). (A) Cytosolic and nuclear fractions from GMs (P) and control (C) macrophages were immunoblotted for p65‐NF‐kB (NFKB) after treatment with lipopolysaccharide (LPS) (100 ng) and supplementation with 3MA (5 mm) and/or rapamycin (25 nm). GAPDH and histone were used as the standards for the cytosolic and nuclear fractions, respectively. Normalized expression levels are shown in the graphs, representing four independent experiments. (B) Nuclear and cytosolic fraction from control (C) and GMs (P), before and after LPS, ATP, and 3MA treatment, were probed for p62. Data represent three independent experiments. (C) Control (C) and GMs (P) were treated with LPS (100 ng) and ATP (5 mm) in the presence and absence of Baf.1A (10 μm) and were stained for p65‐NF‐kB (red) and p62 (green). Images represent 30 pictures taken in 6 independent experiments (63× magnification, scale bar; 5μ). Insets show higher magnification of areas outlined (a, b). (D) Control and GMs treated with LPS (100 ng) alone and supplemented with rapamycin (25 nm) or Baf.1A (10 μm), were stained for p65‐NF‐kB (green) and Lamp2 (red). Images represent 15 pictures from four independent experiments. Insets illustrate co‐localization of NF‐kB with DAPI, a nuclear marker.

As elevated levels of IL‐1β expression were observed in GMs in response to LPS, we focused on p65‐NF‐kB signaling to further evaluate the causes of inflammasome activation in GD. LPS is a known p65‐NF‐kB activator necessary for NLRP3 expression, but often a second stimulus such as ATP is required to activate inflammasomes (Greten *et al*., 2007; Bauernfeind *et al*., 2009). To evaluate p65‐NF‐kB activation, cell fractionation was performed. At baseline, more p65‐NF‐kB was observed in the cytosolic fraction of GMs than in controls, while in the nuclear fraction, p65‐NF‐kB was observed in GMs, but not control macrophages (Fig. 5A). Induction of autophagy using rapamycin after LPS treatment led to less cytosolic p65‐NF‐kB in control macrophages compared to GMs, and again, p65‐NF‐kB was present in the nuclear fraction only in GMs. Inhibition of autophagosome formation using 3MA after LPS treatment led to increased p65‐NF‐kB in both fractions in control macrophages and GMs, although in the pellet, p65‐NF‐kB levels were 2‐fold higher in GMs compared to controls. As regulation of p65‐NF‐kB can occur by autophagy (Chang *et al*., 2013a,b), levels of p62 were measured in the two fractions. Figure 5B shows that in GMs, levels of p62 in both fractions were higher than in controls in the presence and absence of LPS and ATP, even with 3MA treatment. This indicates that in GMs, p65‐NF‐kB recruits the ubiquitin‐binding protein p62 to aggresome‐like structures. Immunostaining of macrophages with p62 and p65‐NF‐kB demonstrated that p65‐NF‐kB translocated from the cytosol to the nucleus in GMs, but not control macrophages, even in the absence of LPS and ATP (Fig. 5C), and co‐localized with p62 in the nucleus. Inhibition of autophagy with Baf.1A after inflammasome activation resulted in increased p62 in the nucleus of GMs only, with strong co‐localization of p62 and p65‐NF‐kB (Fig. 5C). Thus, suppression of autophagy in GMs results in activation of p65‐NF‐kB, IL‐1β secretion and increased expression of inflammatory cytokines. Furthermore, immunostaining for p65‐NF‐kB and Lamp2 (Fig. 5D) demonstrates that in the absence of LPS, there is still more nuclear translocation of p65‐NF‐kB in GMs than in controls. In the presence of LPS, p65‐NF‐kB translocates into the nucleus of macrophages, and no co‐localization of p65‐NF‐kB and Lamp2 was detected in both control and GMs. However, adding rapamycin after LPS treatment resulted in strong co‐localization of p65‐NF‐kB with Lamp2 in control, but not GMs, indicating absent p65‐NF‐kB translocation in control macrophages. In contrast, in GMs, p65‐NF‐kB was present in the nucleus in the presence of rapamycin after priming with LPS. LPS treatment followed by Baf.1A resulted in the transfer of p65‐NF‐kB to the nucleus in both GMs and control macrophages, with no co‐localization with LAMP2 (Fig. 5D). Together, these data indicate that LPS signaling causes translocation of p65‐NF‐kB into the nucleus, and induction of autophagy leads to elimination of p65‐NFkB by the lysosome. However, the impairment of autophagy and lysosomal dysfunction in GMs activate p65‐NFkB, even when autophagy is augmented.

To further explore the consequence of p65‐NFkB activation, expression levels of specific cytokines were measured in control and GMs in the presence and absence of LPS. IL‐10 levels were elevated about 4‐fold in GMs compared to control macrophages, both with and without LPS. In addition, IL‐6 (Libermann & Baltimore, 1990) and IL‐18 (Suk *et al*., 2001) were increased more than 2‐fold in GMs. IL‐12A (Homma *et al*., 2007) and IL‐12B (Murphy *et al*., 1995) levels increased 5 and 7 fold in the absence, and 10‐fold in the presence of LPS (Fig. S4). Thus, the activation of p65‐NF‐kB in GMs is further substantiated by the high levels of inflammatory cytokines.

## Discussion

This study, performed in GMs, demonstrates that impaired autophagy resulting from lysosomal dysfunction interrupts inflammasome homeostasis, as inflammasome turnover is dependent upon functional autophagy. As a result, GMs manifest the activated macrophage (M1) phenotype and secrete inflammatory cytokines including IL‐1β and IL‐6 in the presence of the TLR4 agonist LPS. While one report noted slightly increased IL‐1β by immunohistochemistry in spleens from two patients with GD1 (Boven *et al*., 2004), our finding is supported by studies in patients with type 1 GD showing elevated serum levels of IL‐6 (Allen *et al*., 1997), in mouse models of GD indicating that systemic inflammation is triggered even with minimal glucosylceramide accumulation in macrophages (Mizukami *et al*., 2002) and in iPSC‐derived cells (Panicker *et al*., 2014).

Recent studies have led to an appreciation of the role of autophagy as an elementary form of innate immunity against invading micro‐organisms in eukaryotes (Deretic *et al*., 2013). LPS induces autophagy in macrophages (Sanjuan *et al*., 2007; Xu *et al*., 2007), providing a means to clear invasive bacteria. There are different stimuli for inflammation: a stimulus like the TLR ligand LPS induces expression of pro‐IL‐1β, while activators, like ATP, are required to assemble the NLRP3 inflammasome and initiate the subsequent activation of IL‐1β and IL‐18. Inflammasome stimulation can provoke autophagosome assembly via the ubiquitination of ASC, an inflammasome component, which then recruits p62, an important regulator of autophagy (Shi *et al*., 2012). A direct interaction between p62 and LC3 results in the delivery of inflammasomes to the autophagic pathway (Kraft & Martens, 2012). p62 also promotes the aggregation of ubiquitinated proteins by delaying their delivery to the proteasome and promoting IL‐1β signaling (Korolchuk *et al*., 2009).

Our data show that in human primary GMs, LPS stimulation results in IL‐1β activation. Impaired autophagy in the presence of LPS and ATP leads to constitutive inflammasome activation, as reflected by reduced levels of the autophagy markers LC3II and Atg16L1 and increased levels of p62 in GMs compared to control macrophages. Unlike control macrophages, GMs do not show co‐localization of LC3 and p62 after inflammasome activation (Fig. 3). We hypothesized that in GMs, the impairment is due to a defect in autophagosome maturation, which also results in defective fusion between lysosomes and autophagosomes. We found that treatment with LPS, rapamycin, or 3MA did not significantly affect GCase activity (Fig. S5).

**Figure 6:**
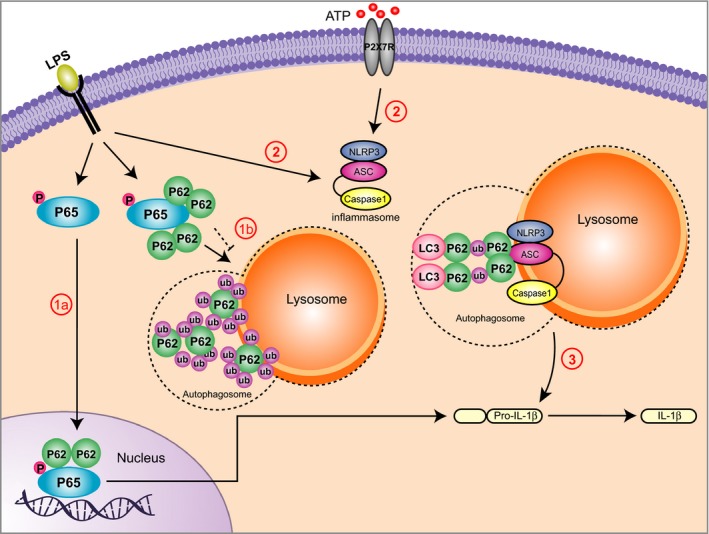
Inflammasome activation due to impaired autophagy in Gaucher macrophages (GMs) (1) In both control and GMs, lipopolysaccharide (LPS) priming induces activation of p65‐NF‐kB, which is translocated to the nucleus, leading to production of pro‐IL‐1β in the cytosol. In control macrophages (1a), LPS stimulates accumulation of ubiquitinated p65‐NF‐kB, which is further recognized by p62, delivered to autophagosomes, and degraded in the lysosome (1a, solid line). In GMs (1b, dashed line), impaired autophagy prevents degradation of p65‐ NF‐kB through autophagy and results in its activation (1b). (2) In both control and GMs, stimulation by both LPS and extracellular ATP leads to inflammasome complex formation (NLRP3, apoptosis‐associated speck‐like proteins (ASC) and caspase1) and activation. (3) Activated inflammasomes undergo ubiquitination of ASC, leading to p62‐mediated engulfment of inflammasomes by autophagosomes. Pro‐IL‐1β conversion to active IL‐1β is limited due to the destruction of activated inflammasomes by autophagolysosomes. In GMs, defective autophagy and lysosomal dysfunction inhibit the elimination of active inflammasomes through autophagy, resulting in the upregulation and secretion of IL‐1β (3).

It has been shown that Atg16L1, in complex with Atg5‐Atg12, suppresses a potent pro‐inflammatory signal by downregulating p62 (Myeku & Figueiredo‐Pereira, 2011). Also, increased IL‐1β activation has been observed in macrophages with dysfunctional Atg16L1, due to enhanced p62 oligomerization (Saitoh *et al*., 2008). The significant reduction of Atg16L1 observed in GMs can be another factor leading to increased SQMT1/p62levels and the inhibition of autophagy in these cells. Autophagy normally regulates inflammation by directing inflammasomes to autophagosomes (Fig. 6). In GMs, no co‐localization of NLRP3 with LC3 was observed after inflammasome activation with LPS and ATP, indicating that this process is disrupted. Moreover, in GMs, caspase‐1 activation was observed after treatment with LPS alone, while in control macrophages, the addition of ATP was also required for caspase‐1 activation.

To further evaluate the impairment of autophagy in GMs, we used rapamycin to induce autophagy. Caspase‐1 was activated even after the induction of autophagy in GMs, but not control macrophages. Caspase‐1 is activated when it interacts with the adapter protein ASC following inflammasome activation. Our data show that ASC is increased in GMs after the induction of autophagy, and co‐localizes with p62, indicating that ASC ubiquitination and its interaction with p62 affects the activation of the inflammasome by inhibiting autophagy. Thus, treating with LPS, or LPS and ATP followed by rapamycin, caused secretion of IL‐1β in GMs, but not control macrophages (Fig. 2E,F).

It is known that p65‐NF‐kB augments the expression of IL‐1β, IL‐6, and other inflammatory cytokines, and is necessary, but not sufficient for NLRP3 activation (Bauernfeind *et al*., 2009). p65‐NF‐kB activation classically occurs via phosphorylation and subsequent degradation of its inhibitor, IkB (inhibitor of NF‐kB), which enables its translocation to the nucleus. Our data clearly show that activation of p65‐NF‐kB in GMs results in increased p62 levels in the nuclear fraction. Furthermore, we observed strong co‐localization of p62 with p65‐NF‐kB in the nucleus of GMs after LPS and ATP treatment. Chang *et al*. (2013a,b) have shown that autophagy targets p65‐NF‐kB to polarize hepatoma‐associated macrophages. Our study indicates that the accumulation of p65‐NF‐kB in the cytosol recruits p62 to form aggresome‐like structures, which are recognized by autophagosomes and then degraded through lysosomes. The inhibition of autophagy in GMs prevents the degradation of cytosolic p65‐NF‐kB and leads to translocation of p65‐NF‐kB to the nucleus (Fig. 6). This, in turn, forces polarization away from alternatively activated macrophages and toward active macrophages (Chang *et al*., 2013a,b). Our data show that induction of autophagy after LPS treatment results in degradation of p65‐NF‐kB via lysosomes, as p65‐NF‐kB co‐localizes with lysosomal markers in control macrophages but not GMs. Hence, we propose that in GMs, increased levels of p62, in both the cytosolic and nuclear fractions, stabilize p65‐NF‐kB in the nucleus, which drives the activation of IL‐1β signaling pathways, resulting in continued activation of IL‐1β and inducing the expression of inflammatory cytokines. Defective autophagy in these cells therefore leads to subsequent activation of both p65‐NF‐kB and the inflammasome.

To further establish the role of GCase in these processes, we used a novel small‐molecule chaperone of GCase to confirm that the observed defect in autophagy and subsequent inflammasome activation is due to lysosomal dysfunction secondary to substrate accumulation. The chaperone, which results in the enhancement of GCase activity and reversal of substrate accumulation^19^, facilitated autophagosome maturation and reduced IL‐1β secretion (Fig. 4E,F), restoring the autophagic capacity of GMs.

In addition to enhancing our understanding of these cellular processes, this study also has significant clinical relevance and provides evidence that GMs manifest inflammatory phenotypes because of impaired autophagy. The inflammation identified may contribute to the tendency toward specific malignancies in some patients, the debilitating bone disease, and potentially the central nervous system involvement in neuronopathic forms of GD. Also, as the vast organomegaly observed in patients with GD cannot be accounted for by the amount of GluCer storage alone, inflammation likely contributes to hepatic and splenic size. The observed failure of fusion of autophagosomes with lysosomes may also be relevant to the recently appreciated association between GD and parkinsonism (Sidransky *et al*., 2009; Siebert *et al*., 2014). In fact, there is literature suggesting that impaired autophagy and lysosomal dysfunction in neurodegenerative disorders can result in a failure to clear accumulated protein aggregates (Lynch‐Day *et al*., 2012; Nixon, 2013). Lysosomal dysfunction is also an important factor in the aging process. Furthermore, our findings provide new targets for the development of therapies to treat GD. Drugs that induce the fusion of lysosomes with autophagosomes may provide a new therapeutic strategy for patients with Gaucher mutations, and may ultimately have implications for patients with parkinsonism.

## Materials and methods

### Collection and genotyping of patient blood samples

Samples from patients with type 1 GD (all with genotype N370S/N370S) were collected with informed consent under NHGRI Institute Review Board‐approved clinical protocols. The age of the patients studied ranged from 40 to 79 years (mean 61 years). Control samples were provided by the NIH Blood Bank. For patients treated with recombinant GCase, samples were collected at least 1 week after their last infusion. Genotypes were confirmed by sequencing all exons of *GBA1,* as described (Stone *et al*., 2000). The number of samples used for each experiment are noted in the figure legends.

### Differentiation of human monocytes into macrophages and reagents used

Peripheral blood mononuclear cells (PBMCs) from controls and patients with type 1 GD were isolated using Ficoll gradients, and monocytes were purified using a CD14 magnetic monocyte enrichment kit (StemCell Technology, Vancouver, Canada). Isolated monocytes were immuno‐phenotyped, and were shown to be CD14+ and CD11b+ (data not shown). Macrophages were differentiated from purified monocytes using 10 ng mL^−1^ of macrophage colony‐stimulating factor M‐CSF (R&D Systems, Minneapolis, MN, USA) in RPMI 1640 medium, supplemented with 10% fetal calf serum (FCS) (Invitrogen, Grand Island, NY, USA). On days 3 and 6, the media were refreshed and macrophages were harvested. Rapamycin (Sigma‐Aldrich, St. Louis, MO, USA) was used at a final concentration of 25 nm, 3MA at 5 mm (Sigma‐Aldrich) and Baf.1A (Tocris, Bristol, UK) at 10 μm. GMs were also treated with 8 μm NCGC758 for 6 days, and media were changed every other day.

For inflammasome stimulation, human macrophages were treated with LPS (Millipore, Darmstadt, Germany) and ATP (Sigma) at a final concentration of 100 ng mL−^1^ and 5 mm, respectively.

### Measurement of activated caspase‐1 and IL‐1β by immunoblotting

Patient and control cells were cultured in 12‐well plates. Fully differentiated macrophages were washed with PBS, and then, 0.5 mL OPTI‐MEM was added along with reagents (ATP, 3MA, Baf.1A or rapamycin). Medium from each well was collected and centrifuged. Five hundred μL of methanol and 125 μL of chloroform were added to 500 μL of collected supernatant. Samples were vortexed and spun for 5 min at 18,000 g at 4 °C. The upper phase was removed and 500 μL of methanol was added, followed by centrifugation at 18,000 g for 5 min at 4 °C. The supernatant was removed, and the pellet was dried for 5 min at 50 °C. The pellet was dissolved in 1:1 NuPAGE^®^ LDS (Life technologies, Grand Island, NY, USA). Sample Buffer and 0.4 m DDT (dichlorodiphenyltrichloroethane) (Shi *et al*., 2012).

### Measuring activated IL‐1β by ELISA

Activated IL‐1β in supernatants from control or patient macrophages subjected to the different treatments were evaluated by ELISA (Affrymetrix, Cleveland, OH, USA) according to the manufacturer's instructions. The IL‐1β concentration in each sample was calculated using the standard curve equation.

### Preparation of Gaucher erythrocyte ghosts

To study autophagy during erythrophagocytosis, macrophages were fed with erythrocyte ghosts prepared from blood samples contributed by patients with type 1 GD as described previously (von der Heul *et al*., 1980; Aflaki *et al*., 2014). Erythrocytes from patients with type 1 GD were used because their membranes are rich in GluCer.

### Quantification of GCase activity in macrophages

Macrophages were cultured in black 96‐well plates. After adding 100 μL enzyme assay buffer (200 mm sodium acetate (pH4), 5 mm 4MU‐βGlu and protease inhibitor cocktail (Sigma), cells were incubated for 1 h at 37 °C. The reaction was halted using a stop solution (1 m NaOH and 1 m glycine) and fluorescence was measured.

### Immunoblotting

Primary macrophages were harvested and sonicated at 4 °C in RIPA Lysate buffer (50 mm Tris‐HCl (pH: 7.4), 150 mm NaCl, 1% NP‐40, 0.5% Na‐deoxycholate, 0.1% SDS, and protease inhibitor). After quantification with BCA (Thermo scientific, Rockford, IL, USA), 10 μg of the lysate was separated by Novex^®^ NuPAGE^®^ SDS‐PAGE Gel System (Life technologies) and transferred to iBlot PVDF membranes (Life technologies). Blots were blocked in 1:1 PBS, Odyssey Blocking Buffer (LI‐COR bioscience, Lincoln, NE) for 1 h at RT. The blocked membrane was incubated in blocking buffer containing 0.1% Tween‐20 and the respective primary antibodies LC3A/B (Cell signaling, Danvers, MA, USA, 1/1000), Atg7 (Cell signaling, 1:1000), histone H3 (Cell signaling, 4499, 1:2000), VPS34 (Sigma, V9764, 1:1000), SQSTM1/p62 (Abcam, Cambridge, MA, USA, 1:1000), β‐actin (Abcam 1:1000), ASC (Enzo life science, Farmingdale, NY, USA 1:1000), caspase‐1 (Enzo life science, 1/1000), NFĸB (Origene, Rockville, MD, USA, 1:500), GAPDH (GeneTex, Irvine, CA, USA, 1:2000), Atg16L1 (Bethyl, Montgomery, TX, USA, 1:2000), cathepsin D (R&D systems, 1:1000), IL‐1β/IL‐1F2 (R&D systems, 1:1000)] overnight at 4 °C, followed by three 15‐min washes, and then was incubated in blocking buffer containing 0.1% Tween‐20 (Sigma), 0.01% SDS, and IRDye^®^, 680RD secondary antibody 1:10 000 (Li‐CORE Bioscience, Lincoln, NE, USA) for 1 h at RT. The blot was imaged using the LI‐COR Odyssey imaging system (Li‐CORE Bioscience, Lincoln, NE, USA) and quantified using imageJ software.

### Cell fractionation

After treatment, macrophages were washed and re‐suspended in NP‐40 lysis buffer (10 mm Tris, pH 7.9; 140 mm KCI; 5 mm MgCl_2_; 1 mm DTT and 0.5% (v/v) NP‐40). The cells were incubated on ice for 15 min. The pellet was separated by centrifugation at 1000 ***g*** for 5 min and washed twice in NP‐40 lysis buffer. Then, this fraction was lysed in buffer (0.5 Triton X‐100, 0.5% SDS, 10 mm Tris, pH 7.5) and sonicated. The supernatant represented the cytosolic fraction.

### Immunofluorescence staining

Human macrophages were plated on glass chamber slides and, after various treatments, were fixed with 4% paraformaldehyde for 30 min. For LC3 staining, cells were fixed with cold acetone for 10 min. After one‐step washing with PBS, cells were blocked in PBS containing 0.1% saponin, 100 μm glycine, and 2% donkey serum (2 h) followed by incubation with antibodies against LC3B (Sigma), SQSTM1/p62 (Cell signaling), ASC (Enzo Life science), cathepsin D (R&D systems), Lamp2 (Hybridoma, Columbia, MD, USA), p65‐NFkB (Origene) for 2 h. Then, cells were washed with PBS three times for 5 min followed by incubation with secondary antibodies. Cells were mounted with Vectashield plus DAPI (Vector laboratories, Burlingame, CA, USA) and were imaged using a Zeiss 510 META laser scanning microscope (Carl Zeiss microimaging Inc., Germany). Images were acquired using a 60X oil DIC objective. Images also were taken with Z‐stack (0.3–0.8 μ) and were analyzed using imaris software (Zurich, Switzerland) for co‐localization and surface measurement.

### Analysis of LC3 puncta

Both control and GMs macrophages were cultured in chamber slides. After the different treatments, cells were washed twice with 1X assay buffer and stained using a Cyto‐ID Autophagy Detection Kit (Enzo life science, Farmingdale, NY, USA), according to the manufacturer's instructions. Cells were imaged by confocal microscopy using a 40X oil DIC objective.

### Statistical analysis

Statistical analyses were performed using graphpadprism6.0 software (La Jolla, CA, USA). Significance was determined by a Student's *t*‐test. Data from two groups or >2 independent variables were analyzed by one‐way ANOVA (nonparametric test; Kruskal–Wallis test). Data are presented as mean values ± SD. Significance levels between controls and patient macrophages were set when *P* < 0.05(*), *P* < 0.01(**), *P* < 0.001(***) and *P* < 0.05(#), *P* < 0.01(##), *P* < 0.001(###) between different conditions.

## Funding

Division of Intramural Research, National Human Genome Research Institute.

## Conflict of interest

The authors have no conflict of interests to declare.

## Supporting information


**Fig. S1** (A) Control and Gaucher macrophages (N370S/N370S) were immunostained for NLRP3 (red) and LC3 (green) in the presence of LPS (100 ng) then imaged by confocal microscopy. (B) Single channels from Fig. 4A are shown separately. (C) Control and Gaucher macrophages (N370S/N370S) were immunostained for p62 (red) and ASC (green) in the presence of LPS (100 ng) then imaged by confocal microscopy.
**Fig. S2** Punctuate assay was performed in control and GMs (N370S/N370S) in 4 independent experiments in the presence LPS/ATP and bafilomycin A1 (50 cells were counted for each condition).
**Fig. S3** (A‐B) Control and GMs (N370S/N370S) were treated with NCGC758 (8 μm) in the presence and absence of Gaucher erythrocyte ghosts. Total lysates were immunoblotted and probed for LC3 and p62. Graph shows the densitometry analysis from two independent experiments (C) Gaucher macrophages were treated with NCGC758 or Imiglucerase (20 μm) followed by LPS+ATP (5 mm). Total protein lysates were run on SDS‐PAGE and were probed for LC3, p62 and Atg16L1 Graph represents densitometry analysis from two independent experiments. *P* < 0.05(*), *P* < 0.01(**), *P* < 0.001(***) represent significance.
**Fig. S4** Levels of mRNA expression of Il‐10, Il‐6, IL‐12A, Il‐12B and Il‐18 were measured in 10 different control and N370S/N370S Gaucher macrophage samples treated with LPS (100 ng) for 24 h. Data were analyzed using ONE WAY‐ANOVA (nonparametric).
**Fig. S5** GCase activity was measured in control and Gaucher macrophages (N370S/N370S) under different conditions.
