# Supplementary material for: Lysosomal storage and impaired autophagy lead to inflammasome activation in Gaucher macrophages
Source: Aging Cell. 2015 Oct 21;15(1):77–88. doi: 10.1111/acel.12409 (PMC4717273; doi:10.1111/acel.12409)
Supplement: Supplementary file 1 — Fig. S1 (A) Control and Gaucher macrophages (N370S/N370S) were immunostained for NLRP3 (red) and LC3 (green) in the presence of LPS (100 ng) then imaged by confocal microscopy. (B) Single channels from Fig. 4A are shown separately. (C) Control and Gaucher macrophages (N370S/N370S) were immunostained for p62 (red) and ASC (green) in the presence of LPS (100 ng) then imaged by confocal microscopy. Fig. S2 Punctuate assay was performed in control and GMs (N370S/N370S) in 4 independent experiments in the presence LPS/ATP and bafilomycin A1 (50 cells were counted for each condition). Fig. S3 (A‐B) Control and GMs (N370S/N370S) were treated with NCGC758 (8 μm) in the presence and absence of Gaucher erythrocyte ghosts. Total lysates were immunoblotted and probed for LC3 and p62. Graph shows the densitometry analysis from two independent experiments (C) Gaucher macrophages were treated with NCGC758 or Imiglucerase (20 μm) followed by LPS+ATP (5 mm). Total protein lysates were run on SDS‐PAGE and were probed for LC3, p62 and Atg16L1 Graph represents densitometry analysis from two independent experiments. P < 0.05(*), P < 0.01(**), P < 0.001(***) represent significance. Fig. S4 Levels of mRNA expression of Il‐10, Il‐6, IL‐12A, Il‐12B and Il‐18 were measured in 10 different control and N370S/N370S Gaucher macrophage samples treated with LPS (100 ng) for 24 h. Data were analyzed using ONE WAY‐ANOVA (nonparametric). Fig. S5 GCase activity was measured in control and Gaucher macrophages (N370S/N370S) under different conditions. [file ACEL-15-077-s001.pdf]

Figure S1

A

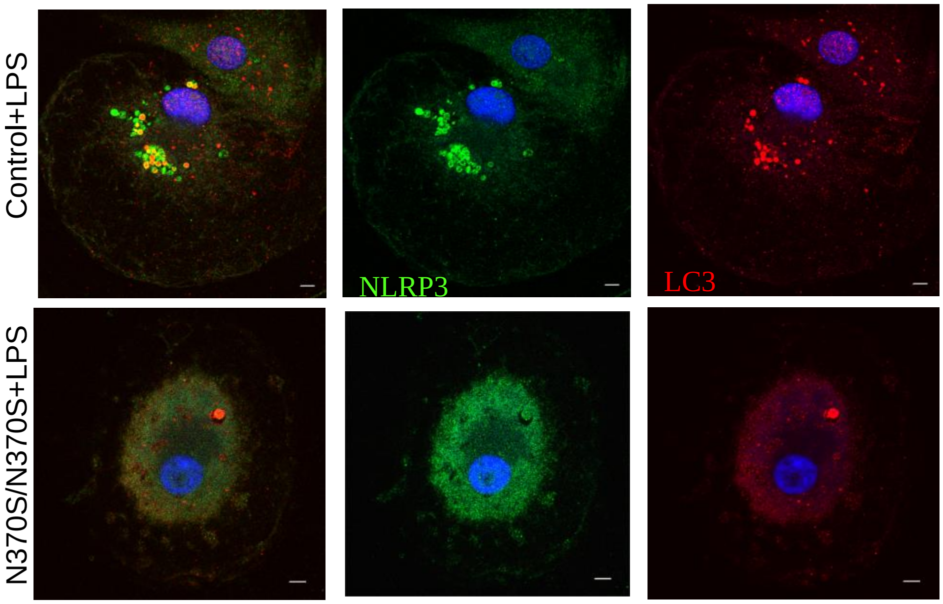
Control+LPS
N370S/N370S+LPS
NLRP3
LC3

B

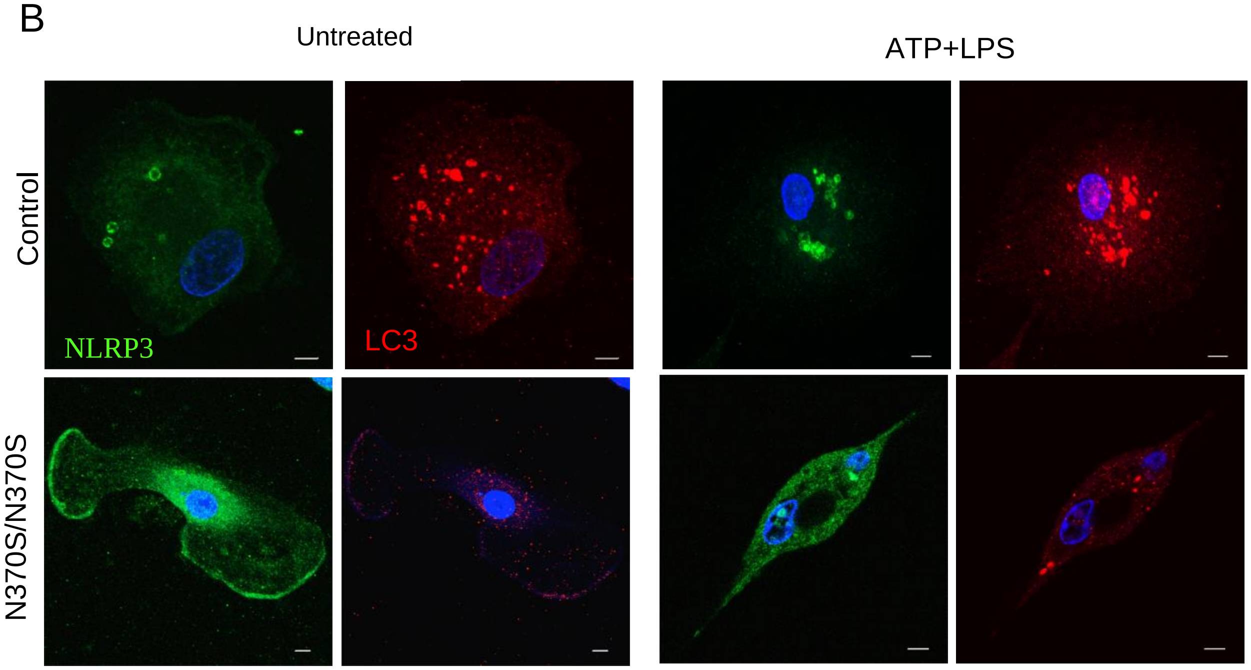
Untreated
ATP+LPS
Control
N370S/N370S
NLRP3
LC3

C

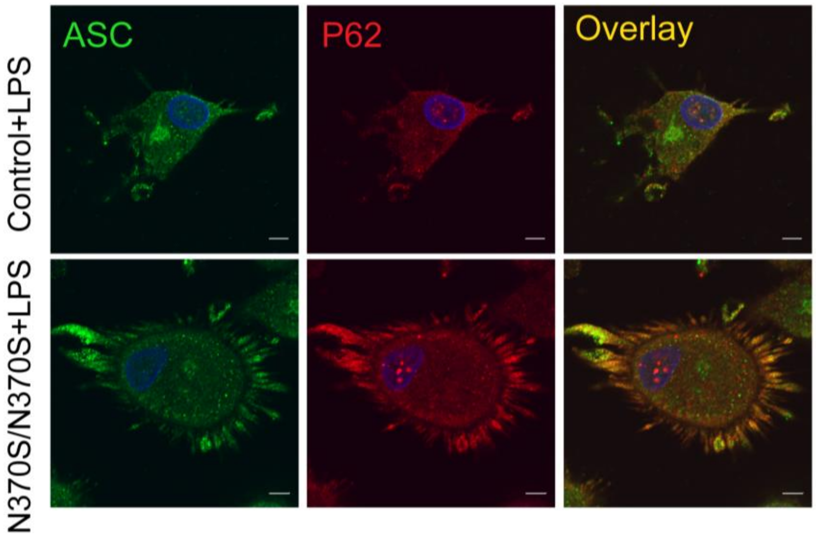

Fig.S1.(A) Control and Gaucher macrophages (N370S/N370S) were immunostained for NLRP3 (red) and LC3 (green) in the presence of LPS (100 ng) then imaged by confocal microscopy**. (B)** Single channels from Fig.4A are shown separately. **(C)** Control and Gaucher macrophages (N370S/N370S) were immunostained for p62 (red) and ASC (green) in the presence of LPS (100 ng) then imaged by confocal microscopy.

Figure S2

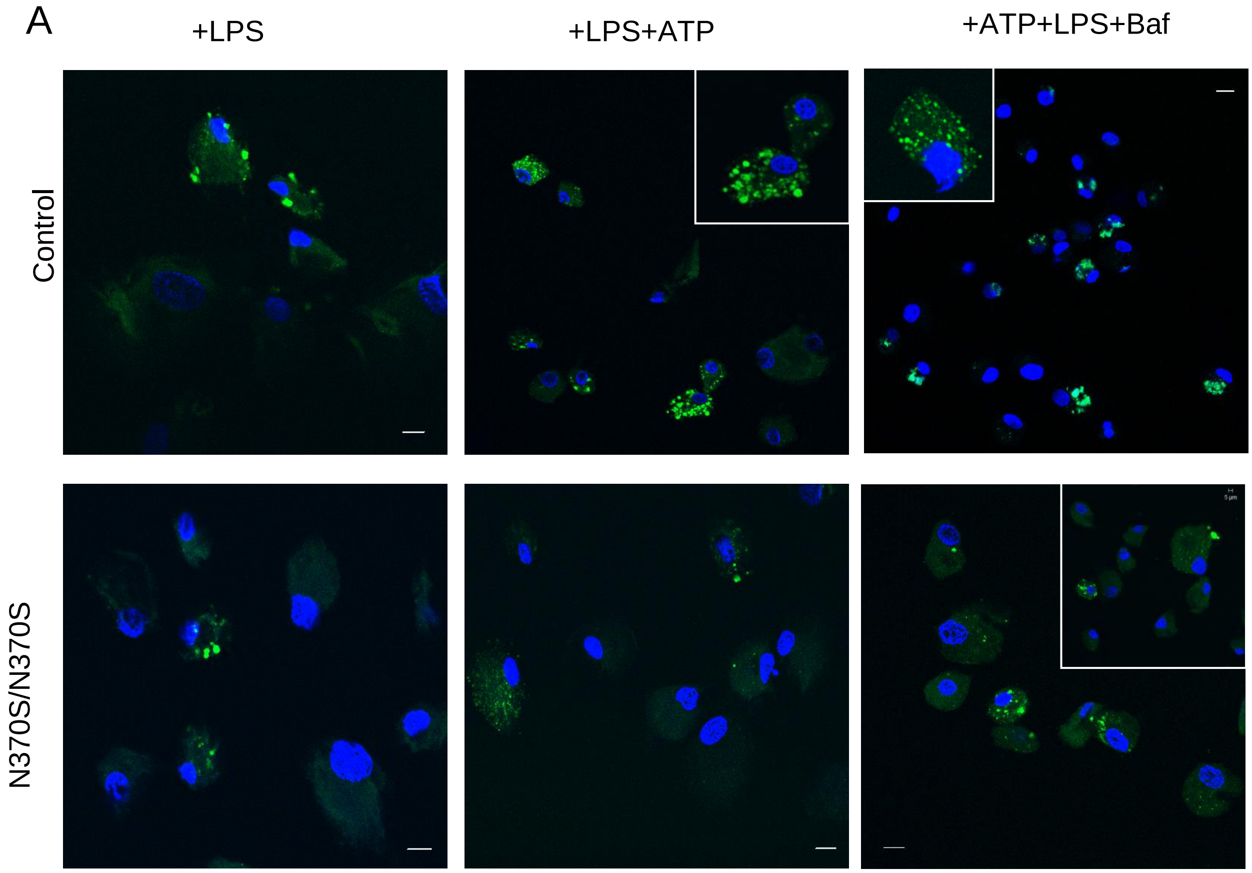

Fig S2. Punctuate assay was performed in control and GMs (N370S/N370S) in 4 independent experiments in the presence LPS/ATP and bafilomycin A1 (50 cells were counted for each condition).

Figure S3

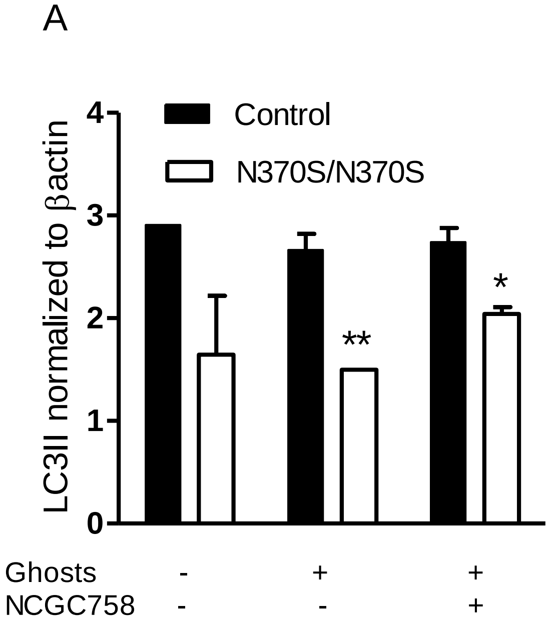

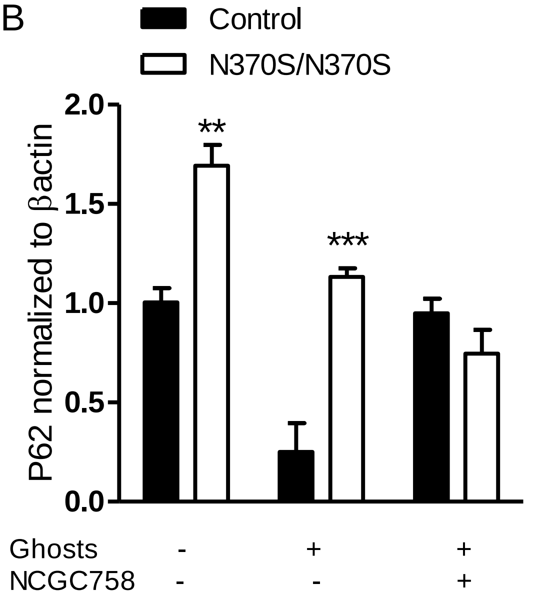

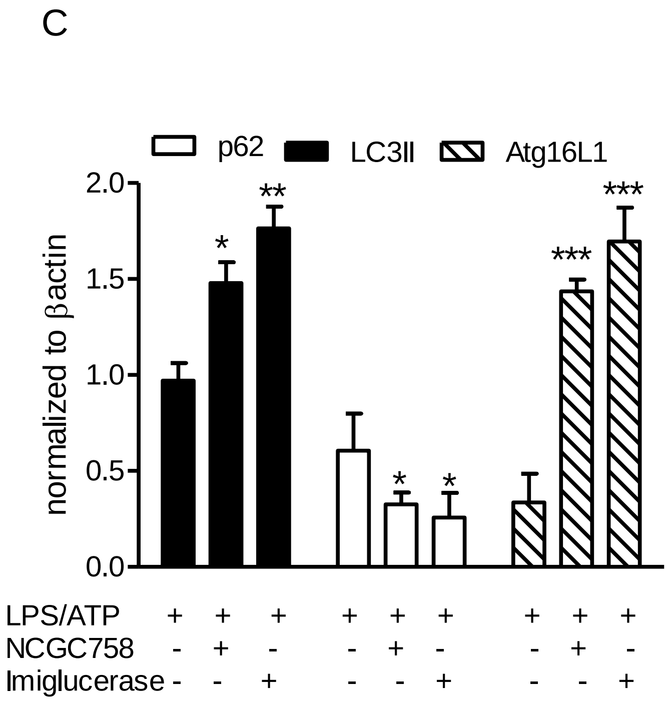

(A-B) Control and GMs (N370S/N370S) were treated with NCGC758 (8 μM) in the presence and absence of Gaucher erythrocyte ghosts. Total lysates were immunoblotted and probed for LC3 and p62. Graph shows the densitometry analysis from two independent experiments **(C)** Gaucher macrophages were treated with NCGC758 or Imiglucerase (20 μM) followed by LPS+ATP (5mM). Total protein lysates were run on SDS-PAGE and were probed for LC3, p62 and Atg16L1 Graph represents densitometry analysis from two independent experiments. $p<0.05$(*), $p<0.01$(**), $p<0.001$(***) represent significance.

Figure S4

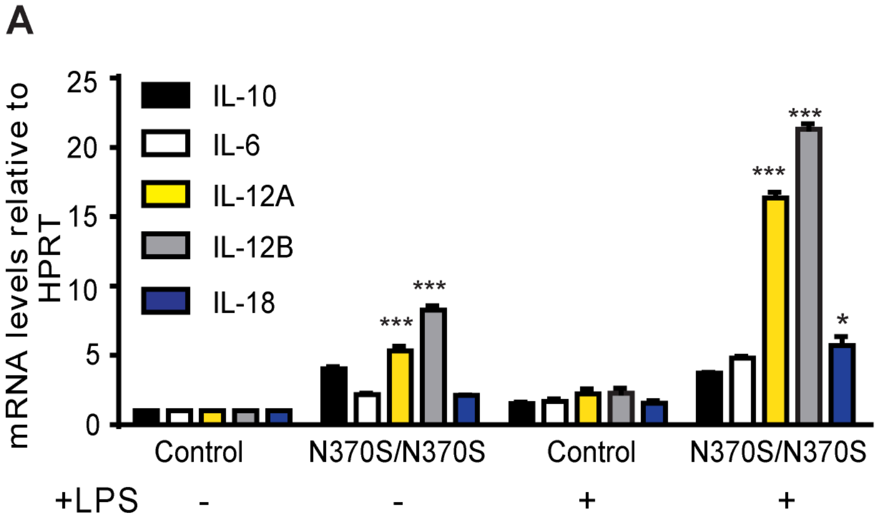

Fig.S4. Levels of mRNA expression of Il-10, Il-6 , IL-12A, Il-12B and Il-18 were measured in 10 different control and N370S/N370S Gaucher macrophage samples treated with LPS (100 ng) for 24h. Data were analyzed using ONE WAY-ANOVA (nonparametric).

Figure S5

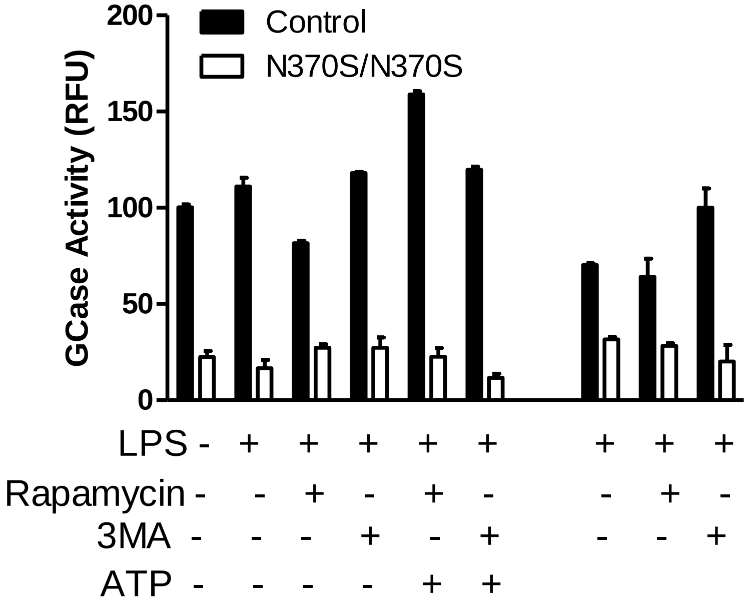

Fig.S5. GCase activity was measured in control and Gaucher macrophages (N370S/N370S) under different conditions.
